# Immunoprotection of Mice against Schistosomiasis Mansoni Using Solubilized Membrane Antigens

**DOI:** 10.1371/journal.pntd.0002254

**Published:** 2013-06-20

**Authors:** Guidenn Sulbarán, Oscar Noya, Beatríz Brito, Diana E. Ballén, Italo M. Cesari

**Affiliations:** 1 Unidad de Trematodiasis, Centro de Microbiología y Biología Celular, Instituto Venezolano de Investigaciones Científicas (IVIC), Caracas, Venezuela; 2 Sección de Biohelmintiasis, Instituto de Medicina Tropical, Facultad de Medicina, Universidad Central de Venezuela, Caracas, Venezuela; 3 Laboratorio de Patología Celular y Molecular, Centro de Medicina Experimental, Instituto de Investigaciones Científicas, Caracas, Venezuela; The George Washington University Medical Center, United States of America

## Abstract

**Background:**

Schistosomiasis continues to be one of the most prevalent parasitic diseases in the world. Despite the existence of a highly effective antischistosome drug, the disease is spreading into new areas, and national control programs do not arrive to complete their tasks particularly in low endemic areas. The availability of a vaccine could represent an additional component to chemotherapy. Experimental vaccination studies are however necessary to identify parasite molecules that would serve as vaccine candidates. In the present work, C57BL/6 female mice were subcutaneously immunized with an *n*-butanol extract of the adult worm particulate membranous fraction (AWBE) and its protective effect against a *S. mansoni* challenge infection was evaluated.

**Methodology and Findings:**

Water-saturated *n*-butanol release into the aqueous phase a set of membrane-associated (glyco)proteins that are variably recognized by antibodies in schistosome-infected patients; among the previously identified AWBE antigens there is Alkaline Phosphatase (SmAP) which has been associated with resistance to the infection in mice. As compared to control, a significantly lower number of perfuse parasites was obtained in the immunized/challenged mouse group (P<0.05, *t* test); and consequently, a lower number of eggs and granulomas (with reduced sizes), overall decreasing pathology. Immunized mice produced high levels of sera anti-AWBE IgG recognizing antigens of ∼190-, 130-, 98-, 47-, 28-23, 14-, and 9-kDa. The ∼130-kDa band (the AP dimer) exhibited *in situ* SmAP activity after addition of AP substrate and the activity was not apparently inhibited by host antibodies. A preliminary proteomic analysis of the 25-, 27-, and 28-kDa bands in the immunodominant 28–23 kDa region suggested that they are composed of actin.

**Conclusions:**

Immunization with AWBE induced the production of specific antibodies to various adult worm membrane molecules (including AP) and a partial (43%) protection against a challenging *S. mansoni* infection by mechanism(s) that still has to be elucidated.

## Author Summary

Schistosomiasis is a neglected disease affecting more than 200 million people globally, especially in sub-Saharan Africa. The mainstay of control of schistosomiasis is Praziquantel, but the mass administration of this drug is unsustainable due to the high rates of re-infection after treatment. These high rates of re-infection point towards the potential emergence of schistosoma drug resistance, making the anti-schistosome vaccine an essential component for the future control of schistosomiasis, as an adjunct to chemotherapy. Multiple strategies have been used to develop an anti-schistosome vaccine with different levels of success. These studies found that the tegument is the most important source of protective antigens; a logical assumption considering this structure represents the surface where the parasite and host interact. In our laboratory, we have isolated a (glyco)protein extract (AWBE) from the whole membrane fraction of adult worms, which is enriched by enzymatic and somatic antigens. Some of these antigens are recognized by infected patients and by mice immunized with irradiated cercariae. Given this context, we tested the possible protective effect of AWBE in mice. The results showed that immunization with AWBE induced a strong humoral response (IgG) with 43% protection against a challenge infection. The AWBE-vaccinated mice showed specific recognition of epitopes in identified proteins, such as schistosome phosphatase and probably actin, pointing to a possible association of these antigens with immunoprotection. These antigens may join the gallery of candidate proteins for vaccination against the infection by schistosomes.

## Introduction

Schistosomiasis is still one of the most prevalent and serious parasitic diseases worldwide; over 200 million persons are currently infected in endemic areas, over 85% of which live in sub-Sahara Africa [1], [2]. Praziquantel (PZQ) remains the main anti-schistosome drug for treatment [3]; however, mass drug administration on *Schistosoma mansoni*-infected populations do not prevent re-infection and patent infections maintain the foci of transmission in low transmission areas [4]. In addition, the repeated use of chemotherapy can result in other complications such as occurrence of drug resistance. Schistosomes are complex organism which sufficient genetic variability to evolve by artificial selection with PZQ [5]. Indeed, schistosomes strain resistance to PZQ has been shown in the laboratory in as few as six generations [6].

In this context, the advent of a schistosomiasis vaccine would be a significant addition to current methods of control of this disease, particularly because in the case of schistosomiasis, a sterilizing vaccine is not essential and partial reduction in worm burdens could reduce morbidity, pathology and limit parasite transmission [7]. One of the main targets of study in the development of an effective vaccine against *S. mansoni* is the parasite tegument [8], a dynamic structure involved in nutrition, excretion, sensory reception and where many different immunoevasion mechanisms and protective-inducing antigens reside [9]–[11].

It has been long since it was demonstrated that adult worm membrane antigens induce antibodies capable of killing the schistosomulum *in vitro* [12]; on the other hand, *in vivo* immunization with tegumental antigens induces partial protection [13]. In the irradiated cercariae model, the production of IgG antibodies recognizing various surface membrane antigens is stimulated; passive immunization with these antibodies induced protection in mice [9]. In the early infection stage newly transformed schistosomula tegument is able to activate dendritic cells and up regulate the expression of co-stimulatory molecules, such as CD40 and CD86, and also to produce IL-12p40 and TNF-α-cytokines [14].

In humans, there is an age-dependent development of immunological resistance to reinfection with *S. mansoni* in population undergoing repeated cycles of infection and treatment [15]. This human resistance is correlated with anti-tegument IgE and IgG antibodies [16]. On the other hand, exposure of cryptic adult tegumental antigens after PZQ treatment is thought to be the key for the success of the anti-schistosoma killing effect of this drug [17]–[19]. One theory holds that upon worm death, either naturally or as a result of treatment, criptical schistosome antigens not normally or appropriately encountered by the host during chronic infection are released [20]. The release of these antigens alters the immune response patterns that results from exposure to intact worm [20], [21] and induce resistance to re-infection [22].

The treatment of a whole adult *S. mansoni* worm membrane fraction (that includes tegumental membranes) with an equal volume of water-saturated *n*-butanol in a two-phase system release several (glyco)components into the aqueous phase that are strongly recognized by IgG antibodies of infected patients, even of patients with low parasitic charges as those living in low-transmission areas [23]. Some of the antigens found in the adult worm *n*-butanol extract (AWBE) are key tegumental surface enzymes like alkaline phosphate (AP), type 5 phosphodiesterase (PDE-5), acid phosphatase (AcP), Ca^2+^-ATPase and acetylcholinesterase (AChE) [24], [25]. AP is recognized by *Schistosoma*-infected human and mouse sera [26]; after treatment with PZQ, AP is exposed on the surface and circulating anti-AP antibodies now inhibit the activity [18].

Based on these results, we considered that the AWBE preparation merited to be tested as possible vaccine antigen in experimental *S. mansoni* infections. Immunization of mice with AWBE induced a strong humoral response and partially protected the animals against a challenge *S. mansoni* infection, meaning that a certain number of parasites could overcome a yet unknown step of elimination and reach maturity. The results contribute to a better understanding of protective anti- adult worm immunity.

## Materials and Methods

### Ethics statement

All animal work was conducted in accordance to the Guide of Care and use of Laboratory Animals published by the US National Institute of Health and adopted by the Committee of Bioethics for Animals (COBIANIM) at the Venezuelan Institute for Scientific Research (IVIC). All protocols for this project were approved by COBIANIM under the approval reference n° 252012. Animals had free access to water and food, under normal daily-night cycle and optimal welfare conditions; when injected, they were carefully handled and excess anaesthesia was used prior to sacrifice.

### Adult worm *n*-butanol extract (AWBE)

Adult *S. mansoni* (Venezuelan JL strain) worms were obtained by perfusion of hamsters infected with 400 cercariae 7 weeks before, washed in sterile saline, and frozen at −80°C until used. Worms were homogenized in Potter-Elvejhem at 4°C with 50 mM Tris-HCl pH 8.0 (Tris-HCl buffer). The worm homogenate was ultracentrifuged for 2 h at 100,000× *g* and 4°C; the particulate pellet, representing the whole adult worm membrane fraction (AWMF), was resuspended and washed two times with the same buffer and centrifuged again as above. The AWMF was resuspended in Tris-HCl buffer, extracted with an equal volume of water-saturated *n*-butanol, and the mixture shaken vigorously at room temperature; the resulting suspension was centrifuged for 15 min at 14,000× *g* and 4°C. The aqueous phase was recovered and dialyzed overnight against Tris-Mg buffer, pH 8.0 and designated as “adult worm *n*-butanol extract” (AWBE), representing the *n*-butanol extract of an AWMF preparation in which membrane-bound surface components are diluted by all the internal membranes and structures. Protein content was determined according to the method of Bradford (1976) [27], using bovine serum albumin as the standard protein.

### Subcutaneous (s.c.) immunizations of mice

Three groups of 10 mice each were used: (I) AWBE-immunized mice; (II) adjuvant- (ADJ); and, (III) buffer-inoculated mice (B). A protocol of three doses at days 0, 15, and 30 was followed. Each mouse was first *s.c.* immunized with 50 µg AWBE mixed with Complete Freund Adjuvant (CFA); the other doses were administered with Incomplete Freund Adjuvant (IFA). Mice injected only with adjuvant (ADJ) or just with buffer (B) were used as controls. Blood samples were obtained by retro-orbital bleeding, centrifuged 5 min to 15,000× *g*, aliquoted, and sera stored to −20°C until used.

### Challenge infection with *S. mansoni* cercariae

Ten days after the immunizations, mice were percutaneously infected with 150 live *S. mansoni* cercariae using the metallic ring method [28]. Briefly, animals were shaved in the abdominal area, anesthetized with Avertin® and immobilized. The metallic ring was placed in the exposed area and the cercarial suspension was added over the skin by 25 min. The level of resistance induced by the AWBE immunization was confirmed by performing a lethal superinfection with ∼450 live *S. mansoni* cercariae/mouse (10 mice/group; AWBE- *vs.* ADJ-group) carried out 10 days after last immunization [29]. Animal health conditions were monitored daily and surviving animals counted.

### Recovery of adult worms

Animals were sacrificed at week 8^th^ post-infection and their portal system and mesenteric veins perfused according to Pellegrino & Siqueira (1956) [30] to determine the n° of intravascular adult worms. The n° of eggs was also counted in the liver (see below). The relative level of protection was calculated from the n° of adult worms in each mouse group according to Shuxian *et al.* (1995) [31], and expressed as percent protection by applying the following formula:
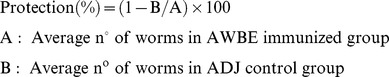



### Measurements of liver weight, volume, and egg number

The liver of each animal was removed and weighed (gr); livers were then placed respectively in a cylinder with 5.0 ml of physiological saline solution and the final volume measured (mL). The egg-induced pathological increase of liver weights and volumes (hepatomegaly) in the different immunizing treatments was assessed by estimating the “excess” weight and volume of each treatment over the liver weight and volume of non immunized control; the average weight and volume measurements of livers in the B control group were assumed to represent the maximum increase (100%) reached by these parameters after *S. mansoni* infection. Percent (%) weight and volume differences between treatments were analyzed by the *t* test (*p*<0.05). To obtain the total number of eggs, dissected livers were completely digested with 4% KOH for 24 h at 37°C [32] and eggs counted in 50-µL aliquots under an optical microscope (10×); the average n° of eggs was deduced from five 50-µL aliquots.

### Histochemistry

After perfusion, livers from each group were dissected and fixed in 10% formalin in 0.1M phosphate buffer pH 7.0 for at least 24 h and maintained in 70% ethanol before being embedded in paraffin. Seven microns paraffin tissue sections of different group mice were stained with hematoxylin and eosin. Briefly, liver tissue sections were deparaffinized in xylene for 10 min, then in consecutive ethanol concentrations from 100% to 80% (v/v), 5 min each. After washing, tissue sections were immersed in hematoxylin for 7 min, washing was repeated and sections placed in a saturated solution of NaHCO_3_, washed again and applied to 95% ethanol. Subsequently, the sections were immersed in eosin, washed in 100% ethanol three times for 3 min, finally immersed in xylene three times for 5 min and store until observed under an optical microscopy.

### IgG response detected by enzyme-linked immunosorbent assay (ELISA)

Microtitulation plates were sensitized with 10 µg/ml of AWBE in 50 mM carbonate/bicarbonate buffer pH 9.6. After overnight incubation at 4°C, three washes were done with PBS-Tween [0.05% Tween, 0.15 M NaCl in 0.01 M phosphate buffer pH 7.4]. Wells were saturated with 4% BSA in PBS (PBS-BSA) for 90 min in a humid atmosphere. Sera (1∶100 in PBS-Tween) were added to the corresponding wells and allowed to incubate for 90 min at 37°C. Wells were then washes 3 times with PBS-Tween and a mouse anti-IgG-Alkaline Phosphatase conjugate (1∶5000 in PBS-Tween) added and incubated for 90 min at 37°C. Three washes were repeated. The Alkaline Phosphatase substrate solution [10 mM *p*-nitrophenylphosphate (*p*-NPP), in DEA/HCl pH 9.6] was added and the reaction allowed proceeding for 10 min at 37°C; the reaction was stopped by adding 1 N NaOH and absorbance readings measured at 405 nm.

### Alkaline Phosphatase Immune Assay (APIA)

Anti-*S. mansoni* alkaline phosphatase IgG levels were determined following the procedure of Cesari *et al.* (1998) [33]. Microtitulation plates were sensitized by overnight incubation at 4°C with 0.5 µg/well of Protein A in 0.1 M carbonate/bicarbonate buffer pH 9.5. Wells were washed 3 times for 3 min each time with Tris-buffered saline (0.01 M; pH 8.2) containing 1% Tween 20 (TBS-Tween) under constant agitation. The wells were saturated with 1% (w/v) casein in TBS-Tween 1 h at 37°C. Washings with TBS-Tween were performed again. Serum (diluted 1∶100 in TBS) was added and the plates incubated for 1 h at 37°C. After washing three times for 3 min with 0.05% Tween-TBS, the plates were allowed to dry at room temperature. AWBE antigen (10 µg/well) was added and plates incubated for 90 min at 37°C. A wash for 3 min was realized with TBS-Tween and other with 10 mM MgCl_2_. The presence of anti-alkaline phosphatase antibodies was detected indirectly by revealing the activity of the SmAP molecules captured by immobilized Protein A-IgG antibodies upon addition of 10 mM *p*-NPP in 50 mM DEA pH 9.6 containing 10 mM MgCl_2_. The enzymatic reaction was stopped adding 1 N NaOH and the absorbance measured at 405 nm.

### Electrophoresis and Western Blot (WB)

Homogeneous non reducing one-dimensional sodium dodecyl sulfate-polyacrylamide gel electrophoresis (1D SDS-PAGE) at 15% (wt/vol) was carried out as described (Laemmli 1970) [34] using a Mini-Protean II electrophoresis chamber. AWBE components (30 µg/lane) were separated at 200 V (constant). Prestained molecular weight standards were run simultaneously on the same gel. AWBE polypeptides were then electrophoretically transferred onto nitrocellulose [35] (NC, 0.45 µm; Hybond ECL); in a semidry blotter at 15v by 15 min a room temperature in 25 mM Tris, 192 mM glycine, and 20% methanol. Free-reacting sites on NC were blocked with 5% skimmed milk in TBS-Tween under constant agitation for 2 h at room temperature. NC strips were cut (2 mm wide), and individual strips were incubated at room temperature under constant agitation for 90 min with the respective serum sample diluted 1∶100 in blocking solution. The strips were next washed with Tris-buffered saline containing 0.05% Tween-20 to remove unbound serum components and then incubated for 90 min at room temperature with a secondary antibody [anti-mouse immunoglobulin G (IgG) peroxidase conjugate] diluted 1∶5000 in blocking solution. Immune reactions were detected by autoradiography on Hyperfilm and using a peroxidase chemiluminescent substrate (ECL detection system). Films were scanned and molecular weights of antigens calculated.

### Detection of alkaline phosphate activity on gels

Separation of the AWBE compounds was carried out in homogeneous non reducing 1D SDS-PAGE at 7% (wt/vol) using minigels as above. At the end of the electrophoretic run, the AWBE gels were placed in an enzymatic activation buffer containing 100 mM NaCl, 5 mM MgCl_2_ in 100 mM dietiletanolamine pH 9.5 for 5 min at 37°C. Immediately thereafter, the gels were incubated in the same buffer containing 0.3 mg/ml of nitrobluetetrazolium (NBT) and 1.65 mg/ml BCIP (5-bromo-4-chloro-3′-indolyphosphate *p*-toluidine salt), the colour produced by the enzymatic hydrolysis of BCIP by AP was allowed to develop in darkness at 37°C; the reaction was stopped by adding 100 mM Tris/HCl pH 8.0 containing 25 mM of EDTA [24], [26].

### Multiple Antigen Blot Assay (MABA)

This is a dot blot technique that allows the simultaneous analysis of different synthetic or natural epitopes from different parasite protein antigens [36]. Mice plasmas were incubated with epitopic synthetic peptides of the following known *S. mansoni* antigens: paramyosin, cathepsin B, glutathione-S-transferase, asparaginyl endopeptidase, and triose phosphate isomerase. The peptides (10 µg/ml) were absorbed individually in parallel rows onto a nitrocellulose (NC) membrane. Afterwards, 2 mm-wide non-fat milk blocked-strips were cut perpendicularly to the peptide-sensitized lines so as to simultaneously expose all peptides to the mouse group sera (1∶100), followed by addition of anti-mouse IgG-horseradish peroxidase (1∶2000); NC membranes were developed with the ECL detection system and Hyperfilm.

### Statistical analysis

Statistics tests used to compare significance results differences between groups was the *t*-paired two-tailed Student's Test. Differences were considered significant when the *p* values were less than or equal to 0.05.

### Accession numbers

Accession numbers [http://www.uniprot.org] [gene, protein]: *Schistosoma mansoni* alkaline phosphatase [EU040139, A8TKU6], *Schistosoma mansoni* type 5 phosphodiesterase (SmNPP-5) [XP_002577090.1., XM_002577044.1., C4QCZ2], *Schistosoma mansoni* acid phosphatase [CABG01000188, C4Q6M8], *Schistosoma mansoni* Ca^2+^- ATPase [AF074400, O96527], *Schistosoma mansoni* acetylcholinesterase [AF279461, Q71SU7], *Schistosoma mansoni* paramyosin [SCMPMYA1, P06198], *Schistosoma mansoni* cathepsin B [SCMCTSB, P25792], *Schistosoma mansoni* glutathione S-transferase [X05148P09792], *Schistosoma mansoni* asparaginyl endopeptidase [AJ250582, Q9NFY9], *Schistosoma mansoni* triose phosphate isomerase [M83294, P48501], *Schistosoma mansoni* actin-1 [XP_002575979, XM_002575933.1, P53470], *Schistosoma mansoni* actin-2 [XP_002578518, XM_002578472.1, P53471].

## Results

### Protection of C57BL/6 mice against a standard and lethal challenge infection with *S. mansoni* cercariae after immunization with AWBE

**Figure 1:**
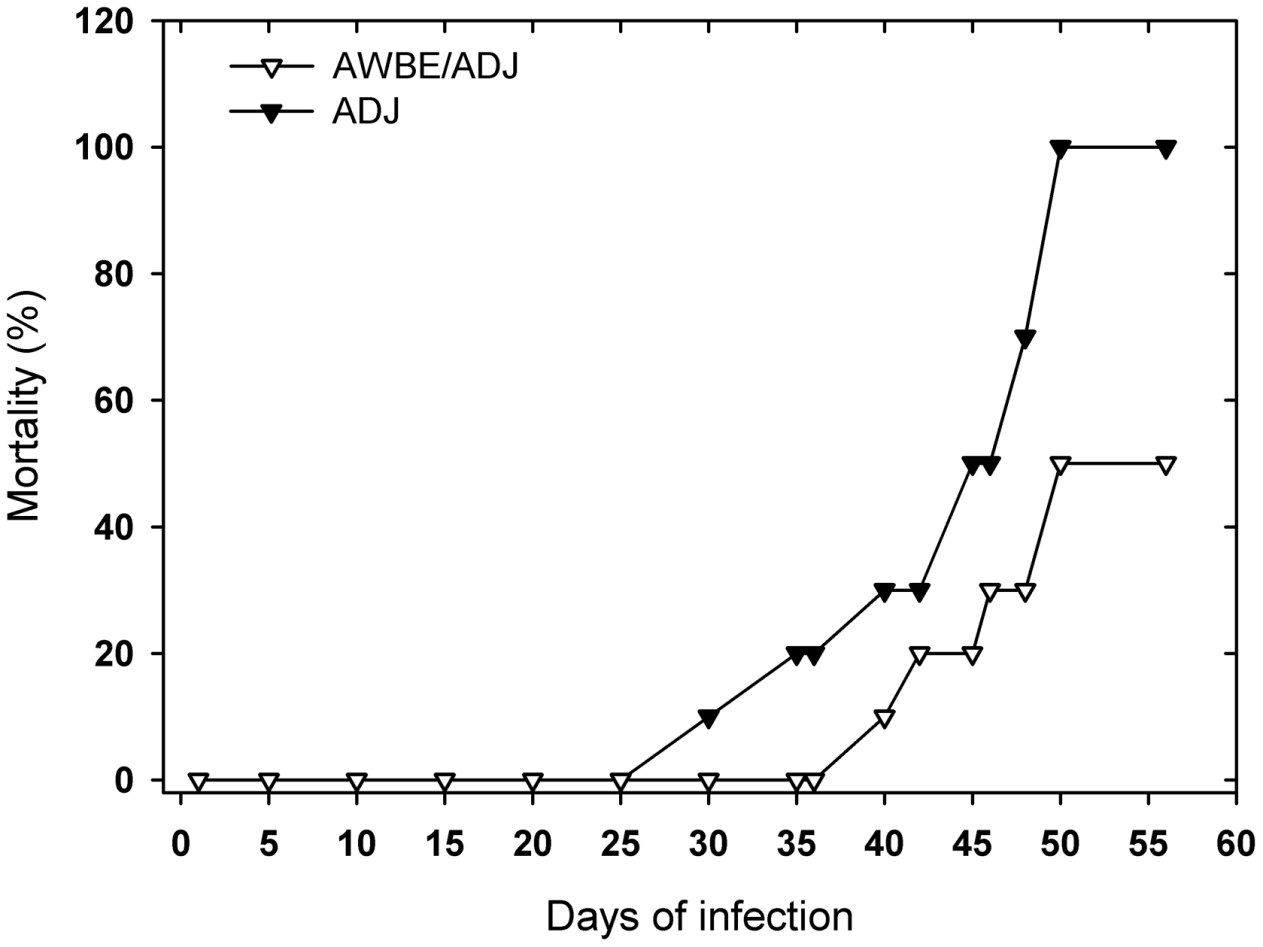
Mortality in C57BL/6 mice immunized with AWBE/ADJ and infected 5 days later with a high parasitic load (∼450 live *S. mansoni* cercariae, JL strain).

The number of *S. mansoni* adult worms obtained by perfusion from AWBE-immunized mouse groups challenged with 150 live cercariae/mouse was significantly lower than those obtained from the challenged unimmunized control group (*p*<0.04 in Exp. I; p<0.02 in Exp. II; *p*<0.0009 in Exp. III) (Table 1). Recovered parasites were all mature adult (male and female) worms and the male/female ratio was not altered, suggesting that AWBE immunization did not selectively affect one or the other sex. Reduction (%) in the number of perfused worms in the AWBE group as compared to the ADJ or to the B control groups was significant (*p*<0.05, *t* test) and ranged from 40.7% to 62.4% (*n* = 3, independent experiments) (Table 1). On the other hand, a reduction of 50% in mortality was observed in AWBE-immunized C57BL/6 mice that were lethally challenged with ∼450 live *S. mansoni* cercariae as compared with the control group 50 days post-infection, in which 100% of the animals died (Fig. 1). After 56 days post-infection, the surviving animals of this AWBE-immunized group were sacrificed; a parasitic load of 63.0±15 adult worms and of eggs of 89.4±15.3 eggs in the hepatic tissue was found. As above, recovered adult worms did not show an altered sex ratio. Unfortunately, extended autolysis in the tissues of the dead animals prevented determination of parasite load and eggs counting. The protection observed in both challenged groups was not due to a decreased infectivity of cercariae in the experimental groups (infectivity was ca. 30% for the strain used) and it was thus related to the AWBE inoculation.

**Table 1:** Protective effect of AWBE antigens against a challenge infection with 150 live *S. mansoni* cercariae in C57BL/6 mice.

Group	Immunogen	Total n° AW^1^	Protection (%)	n° AW^1^		n° Eggs (×10^−3^)
				Males	Females	
I	AWBE	14.0±3.51*	62.4	6.83±2.32	7.17±2.32	68.23±20.3
	ADJ	37.2±2.93		18.60±3.61	18.60±2.58	90.05±15.2
	B	32.4±8.10		16.25±4.32	17.80±5.81	85.23±14.3
II	AWBE	29.2±8.15*	27.4	13.50±3.98	15.50±5.19	59.63±18.7
	ADJ	40.2±8,58		19.80±6.96	20.40±5.27	86.30±25.3
	B	38.9±8.10		20.10±6.96	19.00±5.27	82.39±25.3
III	AWBE	22.9±5.94*	40.7	11.20±2.66	11.60±4.20	62.30±19.6
	ADJ	38.6±7.90		21.10±5.17	20.90±5.52	85.93±24.9
	B	42.0±9.89		19.50±6.34	18.10±3.81	82.93±18.8

1 Average n° of adult worms (males and females) recovered in the perfusion fluid of liver and small mesenteric veins from each experimental group (Mean ± SEM, *n* = 10).

The reduction of the parasitic load was calculated according to formula: % Protection = (1−B/A)×100 [31] where A is the average n° of worms in the AWBE-immunized group and, B the average n° of worms in the adjuvant (ADJ) inoculated group (Control). AW: Adult Worms; AWBE: Adult Worm *n*-Butanol Extract; ADJ: adjuvant inoculated mice; B: mice injected with buffer. Significance by *t* student test (compared to group of ADJ-inoculated animals):

* significant (*p*≤0.05).

### Anti-AWBE IgG levels in immunized mice

**Figure 2:**
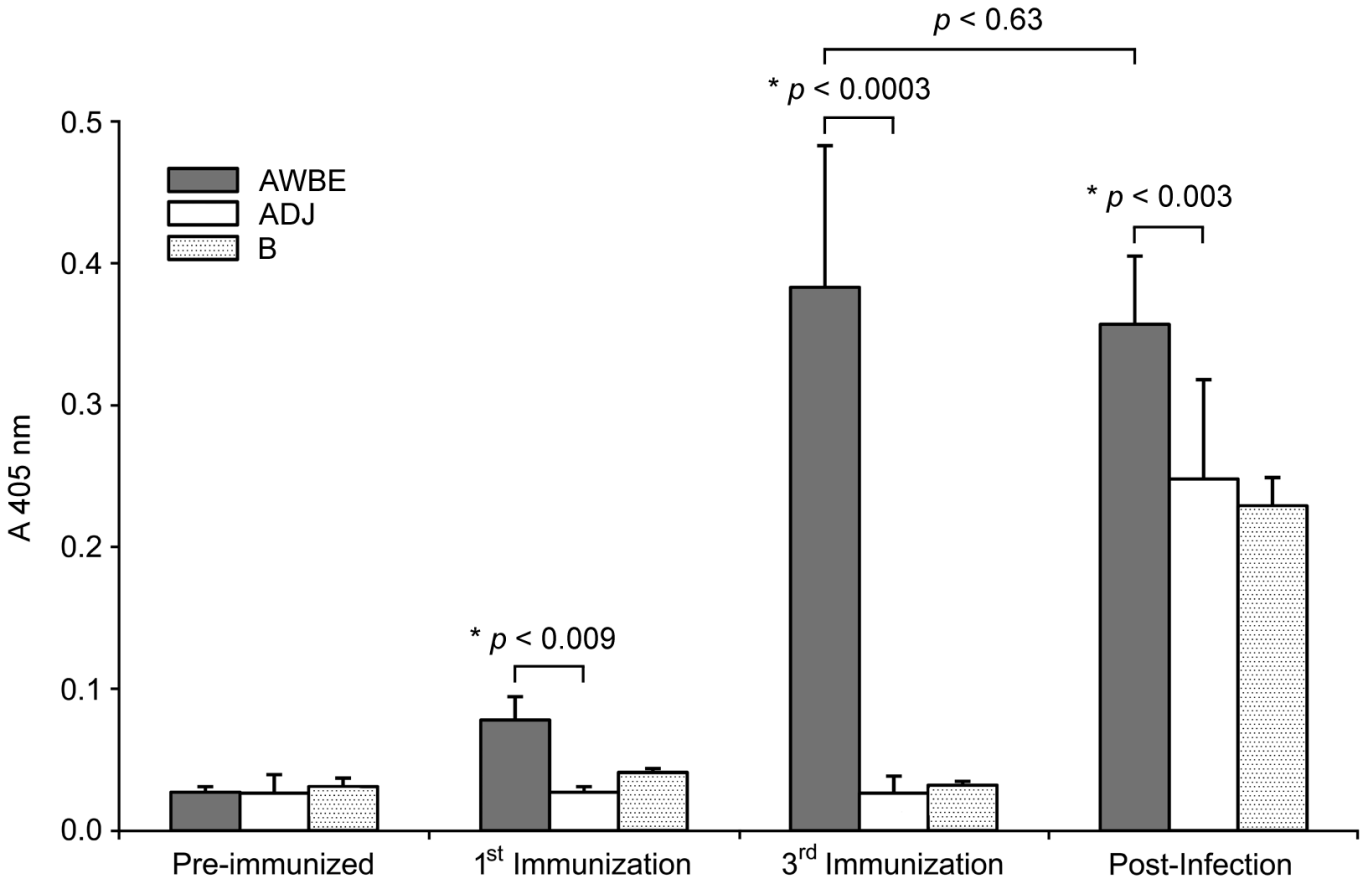
Anti-AWBE IgG antibodies in AWBE-immunized C57BL/6 mice as determined by ELISA. Control groups were represented by mice injected with buffer (B) or Adjuvant (complete and incomplete Freund adjuvant) (ADJ). Results are reported as the mean of three independent experiments ± SEM (*n* = 3). * Significant differences were evaluated by the *t* Student test (*p*<0.05).

Immunization of mice with AWBE produced a significant anti-AWBE humoral response detectable by ELISA from the 1^st^ immunizing dose; this response peaked after the 3^rd^ booster whereas in the control groups (ADJ, B) the absorbance values were not relevant (Fig. 2). However, after the challenge infection, AWBE antigens were recognized by the IgG of control animals (ADJ, B) but at levels significantly lower than AWBE-immunized mice whereas anti-AWBE IgG levels were not significantly altered (Fig. 2).

### AWBE antigens recognized in WB by IgG of the experimental groups

**Figure 3:**
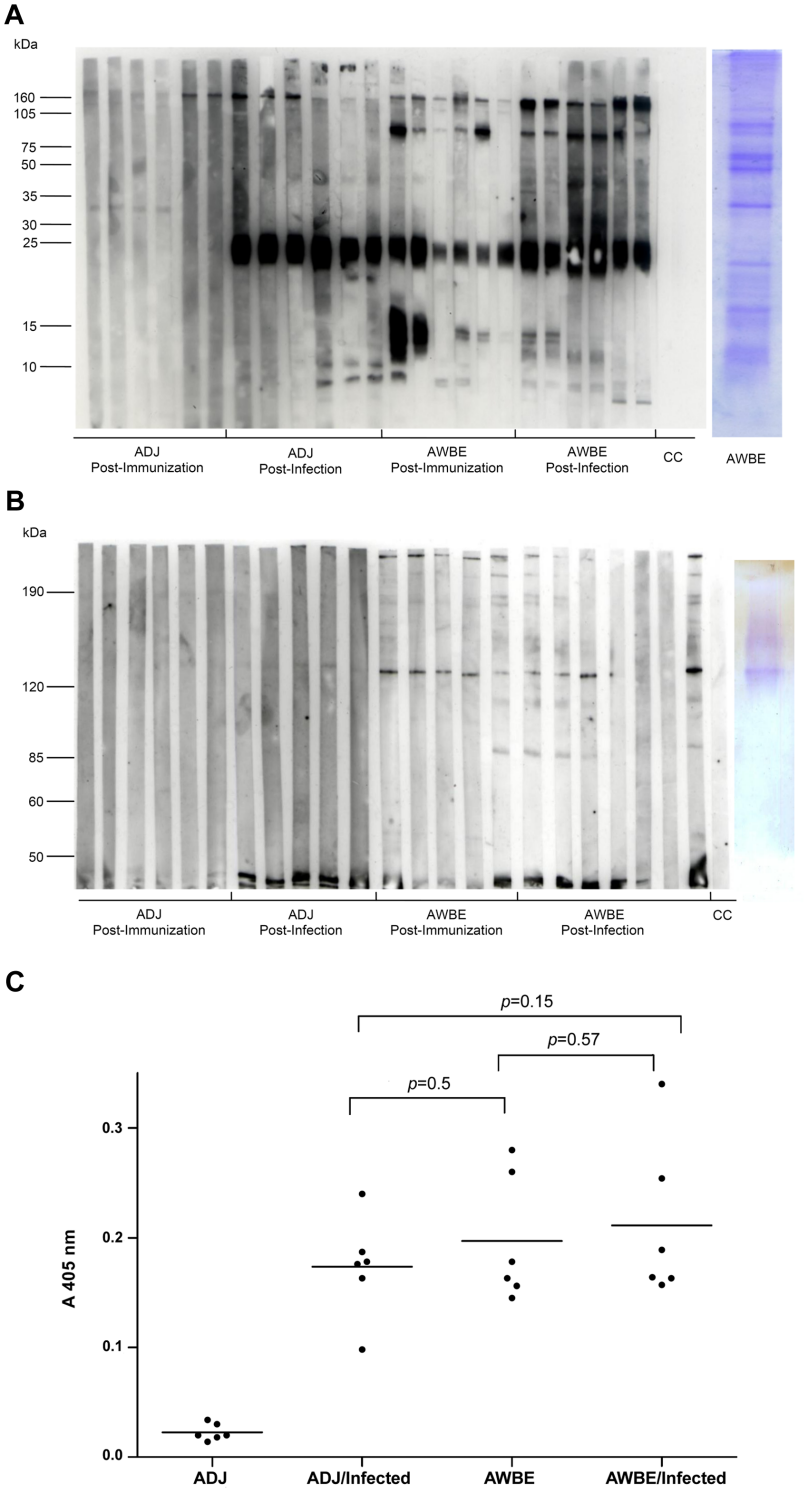
SDS-PAGE/WB analyses of AWBE against sera of treated mouse groups. (**A**) Non-reducing SDS-PAGE (15%, 30 µg/lane)/Western Blot (IgG) profile of *S. mansoni* (JL strain) AWBE against sera from the different mouse groups; on the right, corresponding Coomassie Blue stained 1D gel. (**B**) Non-reducing SDS-PAGE (7.5%, 30 µg/lane)/Western Blot (IgG) profile of *S. mansoni* (JL strain) AWBE against sera from the different mouse groups; on the right: 1D gel stained incubated with alkaline phosphatase substrate (nitrobluetetrazolium/5-bromo-4-chloro-3′-indolylphosphate *p*-toluidine salt) in 100 mM diethylethanolamine pH 9.5). (**C**) Determination of anti-*S. mansoni* (IgG) alkaline phosphatase by APIA. Anti-*S. mansoni* (JL strain) alkaline phosphatase IgG antibodies in sera of the different C57BL/6 mouse groups (6 mice/group). ADJ: adjuvant; AWBE: Adult Worm *n*-Butanol Extract. Mouse groups were exposed to 150 live *S. mansoni* cercariae. Significant differences are denoted by one asterisk * (*p*<0.05, *t* test). Points: individual mice.

Non-reducing 1D SDS-PAGE [(15% Fig. 3A); (7%, Fig. 3B)] of AWBE followed by WB *vs.* mouse sera of the different experimental groups showed differential and specific antibody recognition of a variety of AWBE protein antigens [mainly of M_r_ ∼9-, 14-, 23–28, 47-, 83–93, and −160 kDa (Fig. 3A); 90-, 109-, 130-, and 190-kDa (Fig. 3B)]; however, two bands (33- and 160-kDa) were non-specifically recognized by all the groups, even by pre-immunization sera (Fig. 3A). The M_r_ ∼9-, 23–28, 47-kDa antigens were commonly and specifically recognized by both, immunized/challenged and unimmunized/challenged animals, indicating that AWBE contains molecules that are immunogenic in natural *S. mansoni* infections. On the other hand, the 14-, 83–93 kDa (Fig. 3A) and 130-kDa (Fig. 3B) proteins were more intensely recognized by AWBE-immunized animals. Changes in intensity of recognition of some AWBE antigens were seen in the AWBE-immunized animals after the challenging infection (for example, the 47-kDa band appeared more intense whereas the 14-kDa became more tenuous). Bands composing the 28–23 kDa complex appeared strongly immunodominant after WB (Fig. 3A).

### Alkaline phosphatase (AP) staining

Alkaline phosphatase activity was directly seen in non-reducing 7% SDS-PAGE gels of AWBE when incubated with a specific AP staining; the activity was detected at the level of the 130-kDa band, the expected position for the active SmAP dimer [26]; this band corresponded to the 130-kDa antigen in WBs (Fig. 3B).

### Alkaline Phosphatase Immunoassay (APIA)

Anti-alkaline phosphatase IgG antibodies in the sera of the experimental mouse groups were determined by APIA. APIA values in the non-challenged AWBE-immunized mice did not differ significantly from the APIA values measured after challenge infection (Fig. 3C). On the other hand, IgG from sera of ADJ-inoculated control animals did not recognize the AWBE alkaline phosphatase and were APIA negative, confirming the specificity of the APIA technique.

### Recognition of *S. mansoni* synthetic peptides in MABA

**Figure 4:**
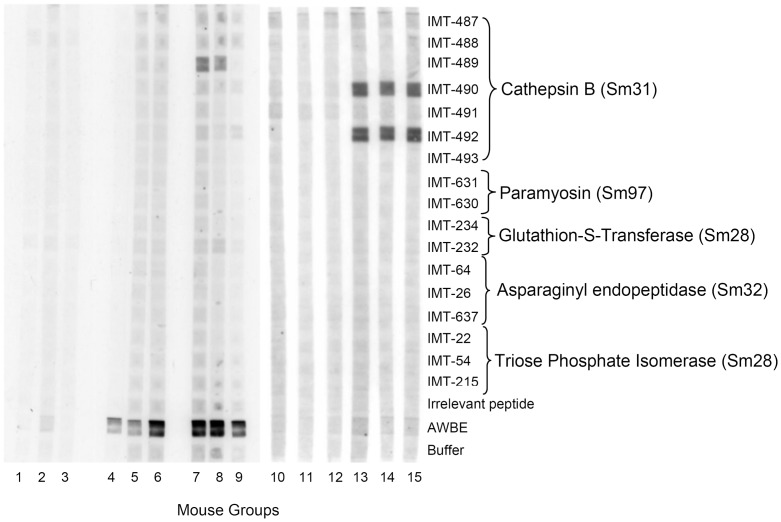
Synthetic peptides sequences derived from known *S. mansoni* soluble protein antigens tested by MABA against sera of the different treated mouse groups. 1–3: Pre-immunization sera; 4–6: sera of AWBE-immunized mice; 7–9 sera of mice immunized with AWBE and then infected with *S. mansoni*; 10–12: sera of mice inoculated with ADJ; 13–15: sera of mice inoculated with ADJ and then infected with *S. mansoni*.

Synthetic epitopic peptide sequences from the known *S. mansoni* antigens Cathepsin B (Sm31), Paramyosin (Sm95), Glutathione-S-transferase (Sm28-GST), asparaginyl endopeptidase (Sm32) and Triose Phosphate Isomerase (Sm28-TPI) (Supplementary Table S1) were tested in MABA against sera from all the experimental groups. None of the peptide sequences was recognized by sera of unchallenged mice. However, some of the *S. mansoni* challenged infected mouse sera, recognized the Sm31 peptide sequences IMT-489 (strong recognition), IMT-490 and IMT-492 (Fig. 4). No anti-AWBE reactivity was observed with sera from mice of the ADJ control group, although those inoculated with AWBE were very reactive against AWBE in MABA (Fig. 4).

### Pathology

**Figure 5:**
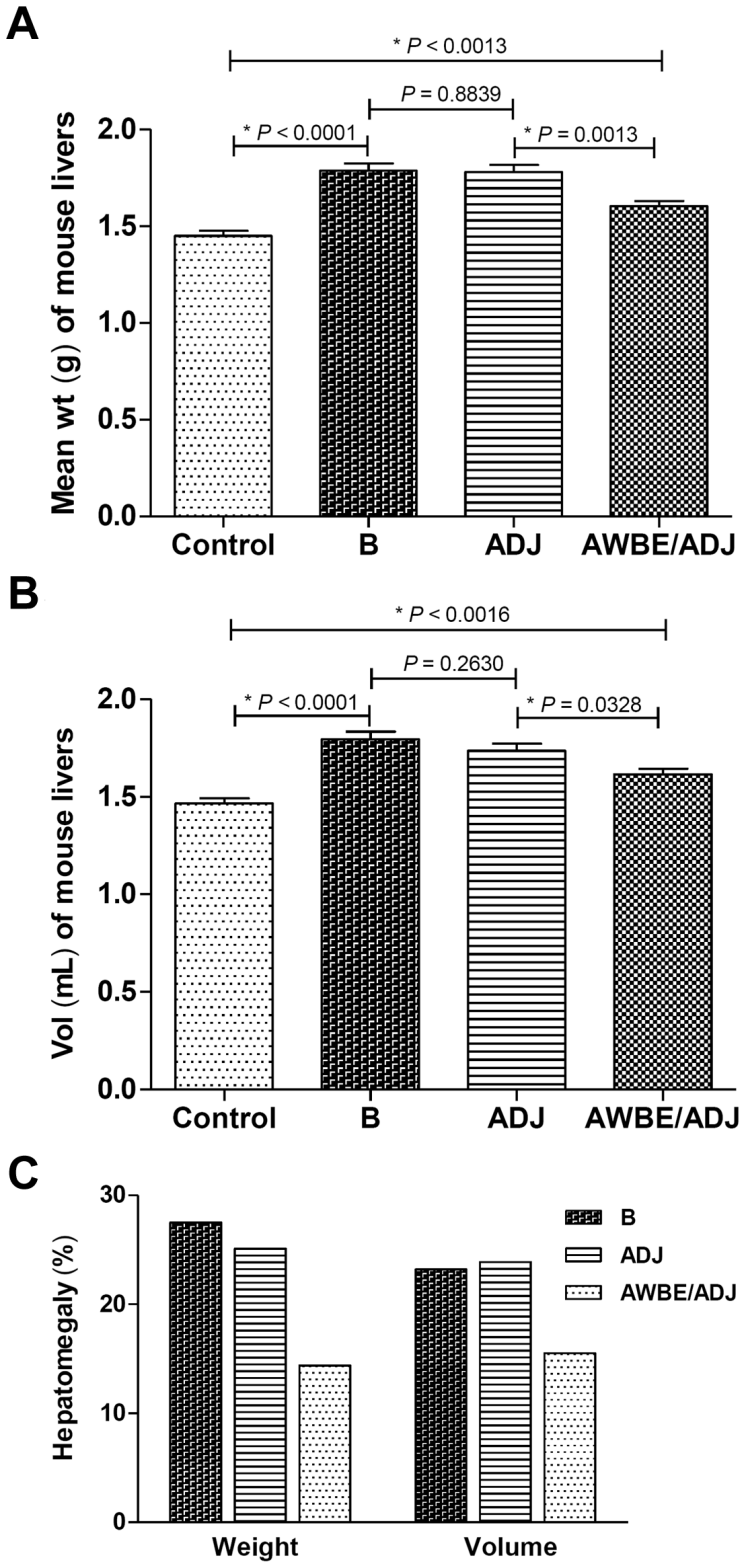
Hepatomegaly measurements of the livers of the different C57BL/6 experimental groups (*n* = 10 per group) after a challenge infection with 150 *S. mansoni* (JL strain) cercariae. Average increase in weight (A) and volume (B) of livers from the differently treated mouse groups after *S. mansoni* challenge infection, in relation to control livers from non-inoculated/non-infected healthy mice, matched for age and sex with mice of the experimental groups. B = buffer; ADJ = adjuvant; AWBE = Adult Worm *n*-Butanol Extract. (C) Hepatomegaly expressed as differential over increase percent (%) of weight and volume in livers of *S. mansoni*-infected mouse groups, compared to absence of hepatomegaly (livers of non-inoculated/non-infected mice).

The degree of inflammatory injury caused in liver by the *S. mansoni* infection in the AWBE-, ADJ- and B-inoculated groups was assessed by measuring the changes in weight and volume of livers (hepatomegaly) in the different groups. As compared to uninfected animals, *S. mansoni* infection altered, as expected, those two parameters, in the B- and ADJ-inoculated groups. However, the partial protection obtained against the challenge infection in the AWBE-immunized mouse group as compared to the ADJ-inoculated group (lower n° of worms recovered after perfusion) (Table 1), was reflected in a lower n° of trapped eggs found in the liver of these mice. Hepatomegaly, as estimated by measuring the increase in weight and volume of the liver, was similar in infected ADJ- and B-inoculated mice, whereas in the AWBE-immunized group, these values were significantly lower (Fig. 5). After infection, livers in the ADJ group incremented their weight in 23.2% and its volume in 22.4%; these values were similar to those obtained in the infected animals of group B (22.7% in weight and 18.3% in volume) (Fig. 5AB). On the other hand, the weight and volume of the livers of the challenged animals that were immunized with AWBE were significantly lower than in the ADJ group; the weight and size increase of livers in this group, as compared to livers from uninfected animals, was 10.5% in weight and 10% in volume (Fig. 5C).

### Histochemistry

**Figure 6:**
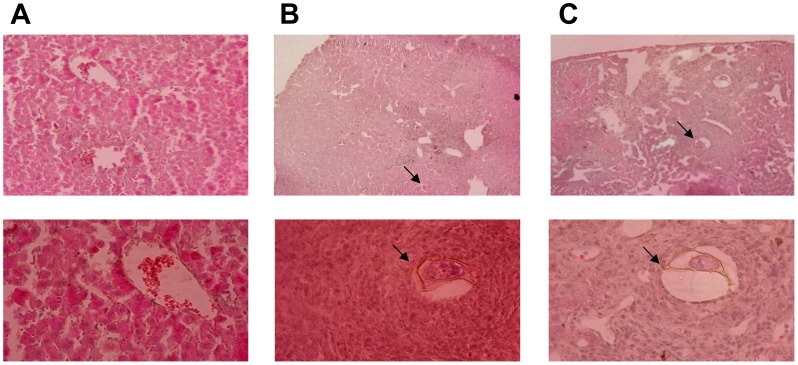
Microscopic appearance of liver in the different mouse groups. Microscopic appearance of histological sections of livers of C57BL/6 mice dissected eight weeks after infection with 150 live *S. mansoni* (JL strain) cercariae. (A) Healthy (250×) (B) Animals immunized with AWBE/ADJ and infected (200×); (C) Animals injected with ADJ and infected (200×). Arrows indicate parasite eggs.

Microscopic analysis of the histological sections of livers from each group revealed that mice of the ADJ-inoculated/challenged group had numerous typical bilharzia granulomas around the parasite eggs (Fig. 6C), while liver sections of AWBE-immunized/challenged mice had less numerous and smaller granulomas (Fig. 6B) and reduced cellularity. In the case of granulomas developed in the AWBE-immunized mice the architecture was less dense and circumscribed, the schistosomal pigment was not present, the liver tissue surrounding the granulomas had less inflammatory infiltration and a better structuring of hepatocytes (Fig. 6B).

## Discussion

Schistosomes, or blood flukes, reside in the hepatointestinal blood vessels of their human hosts. They infect >200 million people in the tropical and sub-tropical world and kill many thousands people each year in developing countries [37]. The adult parasites are surrounded by a unique syncytial layer called the tegument, a structure critical for the parasite survival within the mammalian host; molecules residing in the tegumental membranes are major targets for the development of drugs and vaccines against the parasite [9]. The tegument is delimited by a membrane of multilaminate appearance comprising an inner classical plasma membrane overlaid by an outer external membrane [38]. Relevant proteins like transporters, enzymes and receptors are located in the inner plasma membrane where they are protected from recognition by the host immune system, whereas the outer membrane have a predominant lipid composition with few identified macromolecular constituents that acts like a barrier against the host immune attack [39].

The vigorous mixing of an homogenate of the whole adult *S. mansoni* worm membrane fraction (AWMF) with an equal volume of water-saturated *n*-butanol disrupts the membranous structures favoring the release into the aqueous phase (AWBE) of membrane-bound (glyco)components, particularly enzymes and molecules anchored through glycosylphosphatidylinositol (GPI) linkage to the surface membrane; membrane proteins having a high degree of glycosylation resist denaturation by *n*-butanol, preserving their structure as well as their catalytic function and antigenicity [23], [26], [40]. The *S. mansoni* alkaline phosphatase (SmAP) is an example; this enzyme glycoprotein has been widely accepted and used as a adult worm tegument surface marker [24], [41], [42], [43], [44], and its specific activity is usually enriched 2–4 times in AWBE as compared to the whole membrane fraction (AWMF) (data not shown). Other tegumental components such as Type V Phosphodiesterase (PDE-5 or SmNNP-5) [45], [24], acid phosphatase, Ca^2+^-ATPase, and acetylcholinesterase [23], [24], [46] have been also found in AWBE (Table S3); on the other hand, no proteolytic activity have been so far detected in this preparation (unpublished). These AWBE features prompted us to test the immunizing/protective potential of AWBE in experimental animals.

The subcutaneous (*s.c.*) inoculation of C57BL/6 mice with low doses (50 µg) of AWBE twenty-one days previously to a challenge infection with 150 live *S. mansoni* cercariae per mouse induced a partial protection (∼43% in average) against the challenge infection as compared to non-immunized mice. The level of infection was assessed by counting the n° of adult worms recovered after portal vein perfusion; adult worms were apparently mature. This level of protection was comparable to the most promising results of vaccination against schistosomiasis using tegumentary antigens such as tetraspanins (Sm-TSP-2 or tetraspanin D) [8], Sm29 [47] and Sm80 [48], and recently, GPI-anchored surface antigens [49]. Protection induced by AWBE inoculation was clearly confirmed in the lethal challenging superinfection of C57BL/6 mice where the animals were infected with three times more cercariae (∼450 live *S. mansoni* cercariae) than in the standard experiment, with about 50% of the vaccinated mice surviving the lethal infection as compared to 100% death in control mice. Recovered adult worms did not show an altered sex ratio excluding that percent survivals could be attributed to a unisexual host immune attack.

It was not clear how and where a significant proportion of the challenging parasites were eliminated and how the remaining schistosomes continued to growth apparently unaffected by the immune status of the vaccinated animals. As would be expected, the lower level of infection in AWBE-immunized mice was accompanied by a reduced pathology, as evidenced by a lower n° of eggs found in their livers; there was also a smaller size of the granulomas, lower density (more distance between them), and less liver inflammatory cellularity. Consequently, there was an overall lower increase in weight and volume (hepatomegaly) of the immunized animal livers. There was also absence of the schistosomal pigment in the livers of the immunized animals (which in high worm burdens is a typical result of host hemoglobin degradation by the parasites) [50]. Apparently, immunization with AWBE did not alter the oviposition capacity of the surviving parasites, and AWBE antigens did not exacerbate the inflammatory granulomatous reaction, indirectly confirming that AWBE antigens do not cross-react with egg antigens, as do some soluble adult worm antigens [9].

AWBE contains a mixture of released membrane components, some showing strong antigenicity (28–23 kDa complex). The humoral response against AWBE was characterized by the presence of seric antibodies (IgG) with variable intensity recognition against its components. As an hypothesis, partial resistance to the challenging parasites could be due to an alleatory immune elimination of migrating young parasites by the action of some anti-AWBE antibodies acting against antigens characterized by their early and transitory surface expression in younger forms of parasites and that may be hidden (or not easily accessible) in adult worm membranes; thus, a certain n° of parasites could have been eliminated in early stages of the infection before differentiation into adult forms. Antigens could be present in the tegument of the adult worms that would not be normally accessible to pre-existing circulating antibodies, unless the tegument has been damaged. This is the case in PZQ or Oxaminiquine treatments where the tegumental-damaging action of these two drugs exposes adult worm tegument antigens and accelerates the development of immune resistance to reinfection [51]. Adult worm death would be effected by a pre-existing concomitant immune response to an established adult worm population; this response have been built against tegumental and other worm proteins naturally and continuously entering host circulation as a result of normal parasite protein replacement, surface turnover, exosome production, and/or natural death of the parasites [52]. Some of the proteins (or protein fragments) released from membranes by the *n*-butanol treatment could be the same or similar to the tegumental membrane proteins exposed after drug treatment. AWBE show unique antigenic components that are not seen in detergent-solubilized AWMF when compared in non-reducing 1D SDS-PAGE/WB *vs. S. mansoni*-positive sera (data not shown), suggesting that *n*-butanol is probably releasing proteins from membrane detergent-resistant domains, and/or depolymerizing/disaggregating high molecular weight components not able to enter standard SDS-gels.

Recognition of some AWBE components by antibodies present in *S. mansoni*-infected infected patients [23] might be explained by immune response to released membrane epitopes during infection. Anti-AP antibodies circulating in chronically *S. mansoni*-infected mice did not significantly inhibit the AP activity [18], [25] suggesting that the enzyme active site is not exposed to the immune effectors, and excluding that a functional blocking of this key metabolic enzyme could be responsible for the immune damage to the parasite. However, after PZQ treatment, AP and other molecules (Sm28, actin, etc) becomes critically exposed [18], [52]. Pre-existing circulating anti-AP antibodies act synergically with PZQ to destroy adult worms thus increasing its anti-parasite efficiency [53]. Biotinylation experiments has shown that the *S. mansoni* AP is actually located in the interface of the two plasma membranes of the tegument [54] apparently together with the Sm200 and Sm23 components, associated with membranous structures recognized by anti-caveolin antibodies (possibly caveolas) [46]. In addition, by using immuno-electron microscopy, SmAP appears to be located at the edge of surface pits [44]. This location would imply that some regions or domains of the molecule may be more exposed to the host than others, contributing to explain the SmAP epitope recognition differences found between infected and immunized animals [23], [55]. AWBE has been found to be positive for the presence of caveolin (our unpublished results) suggesting that this component can also be released by *n*-butanol treatment.

Araujo-Montoya *et al.* (2011) [56] found that the surface localization of SmAP was quantitatively supported by its enzymatic activity in live *ex vivo* or cultured parasites throughout the life cycle stages. In addition, the fact that cercariae accumulate large amounts of SmAP mRNA, which rapidly translates into protein upon schistosomula transformation, indicates that AP may have an important role during host invasion [56]. The immune response against this protein could alter the parasite survival directly by affecting nutrient uptake [38] or indirectly by affecting the capacity of SmAP to generate anti-inflammatory molecules such as adenosine [44]. On the other hand, SmAP may not expose the same epitopes in schistosomula; indeed, our previous work has shown that an anti-adult SmAP monoclonal antibody did not recognize schistosomula AP [55].

The possibility that anti-AWBE antibodies could be recognizing known *S. mansoni* vaccine protein sequences in AWBE bands having molecular masses similar to the vaccine antigens described in the literature was partially excluded by the MABA experiment. This dot blot technique allowed the simultaneous analysis of different synthetic or natural antigens from different well known parasite antigens [36]. Short synthetic polymeric peptide sequences of cathepsin B (Sm31), paramyosin (Sm95), glutathione S-transferase (Sm28), asparaginyl endopeptidase (Sm32), and triose phosphate isomerase (Sm28) were not recognized by anti-AWBE antibodies triose phosphate isomerase (Sm28) were not recognized by anti-AWBE antibodies discarding the short epitopic sequences (although not necessarily the corresponding whole proteins) shown in Table S1 as responsible for the protective results here obtained with AWBE. However, after infection, AWBE-immunized animals produced antibodies to IMT/489, a synthetic peptide corresponding to position 89–105 of the schistosome cathepsin B sequence (CB2) or Sm31 antigen; this sequence position is the less antigenic region of the cathepsin B molecule since it is not exposed according to its Hopp & Wood hydrophilicity profile [57].

Antibodies in sera of the AWBE- or ADJ-immunized mice did not inhibit the alkaline phosphatase (AP), phosphodiesterase-5 (PDE-5), or acetylcholinesterase (AChE) activities present in AWBE; however, after infection, a non-significant and partial inhibition of the AP and PDE-5 activities was observed with the AWBE-immunized sera as compared to control group sera; minor effects were registered on the AChE (not shown).

A preliminary proteomic analysis (LC/MS/MS) of faint coomassie blue-stained gel bands of ∼28-, 27-, and 25-kDa in correspondence to the strong immunodominant 28–23 kDa complex showed the presence of *S. mansoni* actin-matching sequences in those bands (Sulbarán *et al.*, unpublished; Table S2). Actin is one of the most important structural components that have been identified by proteomics studies in tegumentary [58], [59] and worm vomitus fractions [60]. In schistosomes, actin is a component of the muscular system and, in its paracrystalline form, conform the typical surface schistosome spines [61]. More important, it has been identified in other studies as the PZQ target [17], [20]. Loss of spines is a typical feature of schistosomes obtained from praziquantel-treated infected animals [17]; in addition, these authors consider that PZQ treatment involves exposure of the surface actin-containing spines and a subsequent host response against actin. Disintegration of spines *in vivo* was associated with binding of host antibodies, but also destruction could be seen in the presence of normal human serum due to the Ca^2+^-dependent binding of gelsolin, an actin depolymerizing factor [17]. Schistosome actin has a molecular mass of 42–45 kDa [62]; thus, the 28-, 27-, and 25-kDa actin-containing bands might be possibly originated either by limited proteolysis of actin filaments during the extraction procedure with *n*-butanol [63] and/or more probably by the known depolymerizing effect that *n*-butanol has on homopolymeric structural proteins [63]. Actin is not evidenced in gels of detergent treated membranes [17]; but treatment with *n*-butanol would seem to expose its antigenic epitopes. Actin is a conserved molecule, but the 28–23 kDa complex is recognized by 96% sera of *S. mansoni*-infected patients from low (Venezuela) [23], by sera of *S. mansoni*-infected patients from high schistosomiasis transmission areas (Brazil), and also by sera of mice that were protectively immunized with the irradiated cercarial vaccine (unpublished), supporting a possible relationship between recognition of this complex and protection, in our case by AWBE. Other structural worm muscle-associated polymeric proteins like paramyosin [64] and the C-terminal region (Irv-5) of myosin, showing also highly conserved sequences, have been reported to be schistosome protective antigens [65] and have been also tested as candidates for vaccination [66]. Apparently, the immune-mediated alteration of the worm muscular movement severely decrease the viability of the parasite, an effect that is also generated by the severe and irreversible muscle contraction induced by PZQ [67]. The conserved glycolytic enzyme triose-phosphate isomerase [TPI-Sm28] has been also described as a protective antigens against *S. mansoni* [68]. Mouse immunization with purified TPI-Sm28 induced a partial but significant 38% protection against *S. mansoni* infection [69] despite its sequence showing 79–87% amino acid homology with mammals TPI [70].

As mentioned, an essential point in this model is to identify the host site where a certain percent of the worms were eliminated and by which mechanism. The morphology of the worms recovered from the immunized animals was apparently not affected and not abnormal changes were noticed after microscopic examination, indicating that, if an immune attack took place, it had happened when the migrating parasites where still immature. In mice vaccinated with irradiated cercariae, the delayed migration of the partially damaged larvae allows the elimination of the immature forms of the parasite, the subsequent immune status preventing later reinfection [71]. The lung schistosomulum seems to be the main target of action of protective immune mechanisms induced by the radiation-attenuated vaccine in the mouse model [72], [73].

It has been reported that 28% of the proteome inventory of *S. mansoni* has no sequence similarity with previously identified proteins in this parasite [74]; AWBE contain a set of new solubilized antigenic polypeptide specificities that deserve molecular identification, looking for additional antigens associated with protection, or looking for possible modulators of the immune response. As a corollary of our results, the AWBE vaccination experience allow us to suggest that a mixture of highly immunogenic, hidden or not accessible epitopes and strategic proteins (like alkaline phosphatase, actin, etc.) rather than individual isolated antigens, may be the way to get a vaccine against schistosomiasis; the inclusion in a vaccine preparation of antigens able to induce anti-morbidity antibodies would also help to control egg-mediated pathology, and finally parasite transmission.

## Supporting Information

Table S1 **Synthetic peptides derived from known** ***Schistosoma mansoni*** **protein antigens used for MABA.** Short synthetic polymeric peptide sequences corresponding to cathepsin B (Sm31), paramyosin (Sm95), glutathione S-transferase (Sm28), asparaginyl endopeptidase (Sm32), and triose phosphate isomerase (Sm28) were tested against anti-AWBE antibodies produced by AWBE-immunized as compared to unimmunized animals.
(DOC)

Table S2 **Preliminary mass spectrometry analysis of AWBE bands of ∼28-, 27-, and 25-kDa from the immunodominant 28–25-kDa complex and peptides alignment (BLASTp) to** ***Schistosoma sp.*** **actin sequences.** CBB-stained AWBE bands of ∼28-, 27-, and 25-kDa were scalp-excised from 15% non-reduced SDS-gels in correspondence to the immunodominant 28–23 kDa complex, eluted and analyzed by LC/MS/MS; hit peptides having a >32 score in MASCOT were aligned by BLASTp against reported *S. mansoni* actin-1 and actin-2 sequences.
(DOC)

Table S3 **AWBE-containing components.** Known AWBE components previously identified by their biochemical and/or immunological properties.
(DOC)

References S1 **References to data and contents reported in Tables S1–3 are included in this section.**
(DOC)
