# Supplementary material for: Immunoprotection of Mice against Schistosomiasis Mansoni Using Solubilized Membrane Antigens
Source: PLoS Negl Trop Dis. 2013 Jun 20;7(6):e2254. doi: 10.1371/journal.pntd.0002254 (PMC3688544; doi:10.1371/journal.pntd.0002254)
Supplement: Table S1 — Synthetic peptides derived from known Schistosoma mansoni protein antigens used for MABA. Short synthetic polymeric peptide sequences corresponding to cathepsin B (Sm31), paramyosin (Sm95), glutathione S-transferase (Sm28), asparaginyl endopeptidase (Sm32), and triose phosphate isomerase (Sm28) were tested against anti-AWBE antibodies produced by AWBE-immunized as compared to unimmunized animals. (DOC) [file pntd.0002254.s001.doc]

| SEQUENCES | **PROTEIN** | **MW**  (**g/mol**) |
| --- | --- | --- |
| CGETFLDITEHKRGC | [1] Sm97 | 1837,30 |
| CGLLHINQDYEAQILNGC |  | 2004,50 |
| CGGNLLASSPRLAKYLSNRPATPFGC | [2] Sm28 | 2694,70 |
| CGKPQEEKEKITKEILNGKGC |  | 2333,00 |
| CGRTLDQYKEVKRETDLSHVQGC | [3] Sm32 | 2794,10 |
| CGRDKSSENDEPPMKPRHSIASRGC |  | 2845,60 |
| CGFLKVLKGDSAGGKVLKSGKGC |  | 2381,60 |
| CGLADYSYNWIVDSQTHHLTQGC |  | 2725,30 |
| CGIPSNFDSRKKWPGCKSIATIGC | [4] Sm31 | 2725,8 |
| CGCWSFGAVEAMSDRSCIQSGGKQNVELSAVDGC |  | 3496,3 |
| CGTASSKENHTGCEPYPFPGC |  | 2185,8 |
| CGYNTPRCKQTCQRKYKTPYTQDKHRGKSSYNVKNDEKAIQKEIMKYGC |  | 5867,1 |
| CGYNTPRCKQTCQRKYKTPYGC |  | 3071,6 |
| CGTQDKHRGKSSYNVKNDEKAIQKEIMKYGC |  | 3134,1 |
| CGENGYFRIVRGRDECSIESEVIAGRINGC |  | 3304,3 |
| CGKWFKTNAPNGVDEKIRIIYGGC | [5] Sm28-TPI | 5336,4 |
| CGEVCVRQLKAIANKGC |  | 1792,40 |
