# Supplementary material for: Immunoprotection of Mice against Schistosomiasis Mansoni Using Solubilized Membrane Antigens
Source: PLoS Negl Trop Dis. 2013 Jun 20;7(6):e2254. doi: 10.1371/journal.pntd.0002254 (PMC3688544; doi:10.1371/journal.pntd.0002254)
Supplement: Table S2 — Preliminary mass spectrometry analysis of AWBE bands of ∼28-, 27-, and 25-kDa from the immunodominant 28–25-kDa complex and peptides alignment (BLASTp) to Schistosoma sp. actin sequences. CBB-stained AWBE bands of ∼28-, 27-, and 25-kDa were scalp-excised from 15% non-reduced SDS-gels in correspondence to the immunodominant 28–23 kDa complex, eluted and analyzed by LC/MS/MS; hit peptides having a >32 score in MASCOT were aligned by BLASTp against reported S. mansoni actin-1 and actin-2 sequences. (DOC) [file pntd.0002254.s002.doc]

**______________________________________________________**

**10 20 30**

**Sm actin-1 MAEEDVAALV IDNGSGMCKA GFAGDDAPRA…**

**Sm actin-2 MADEDVQALV VDNGSGMCKA GF-GDDAPRA…**

**28-kDa MADEDVQALV VDNGSGMCKA GF-GDDAPRA…**

**Sj actin MADEEVQALV VDNGSGMCKA GFAGDDAPRA… Sb actin MADEEVQALV VDNGSGMCKA GFAGDDAPRA…**

**------------------------------------------------------**

**190 200**

**Sm actin-1 …RDLTDYM MKI… …RGYS FTTTAERE…**

**Sm actin-2 …RDLTDYL MKI… …RGYS FTTTAERE…**

**25-kDa …RDLTDYL MKI… …RGYS FTTTAERE…**

**27-kDa ……………………………… …RGYS FTTTAERE…**

**28-kDa …RDLTDYL MKI …RGYS FTTTAERE…**

**Sj actin …RDLTDYL MKI …RGYS FTTTAERE…**

**Sb actin …RDLTDYL MKI …RGYS FTTTAERE…**

**------------------------------------------------------**

**320 330**

**Sm actin-1 …RMQKEISA LAPSTMKIKI VAPPERK…**

**Sm actin-2 …RMQKEITA LAPSTMKIKI VAPPERK…**

**25-kDa …………KELTA LAPSTMKI……………………………**

**27-kDa …………KELTA LAPSTMKI……………………………**

**28-kDa …………KELTA LAPSTMKI……………………………**

**28-kDa …RMTKELTA LAPSTMKIKV VAPPERK…**

**Sj actin …RMQKEITA LAPSTMKIKI VAPPERK…**

**Sb actin …RMQKEITA LAPSTMKIKI VAPPERK…**

**______________________________________________________**
