# Supplementary material for: Immunoprotection of Mice against Schistosomiasis Mansoni Using Solubilized Membrane Antigens
Source: PLoS Negl Trop Dis. 2013 Jun 20;7(6):e2254. doi: 10.1371/journal.pntd.0002254 (PMC3688544; doi:10.1371/journal.pntd.0002254)
Supplement: Table S3 — AWBE-containing components. Known AWBE components previously identified by their biochemical and/or immunological properties. (DOC) [file pntd.0002254.s003.doc]

| **Components** | **kDa** | **Function** | **Features** | **References** |
| --- | --- | --- | --- | --- |
|  |  |  |  |  |
| Alkaline Phosphatase  (SmAP) | 130 | Nucleotides and organic phosphates hydrolysis | • GPI-linked  • redox sensitive  • antigenic | [1] [2] [3] |
| Ca^2+^-ATPase | 112 | Ca^2+^ -dependent ATP hydrolysis  Ca^2+^ tolerance | • antigenic | [4] [5] |
| Acetylcholinesterase | 79 | Signal transduction | • GPI-linked | [6] |
| Acid Phosphatase | 54 | Nucleotides and organic phosphates hydrolysis | • antigenic | [4] |
| SmNPP-5 (PDE-I) | 52 | Nucleotides  Organic diphosphates | • antigenic  • GPI-linked | [4] [7] |
| Male-specific antigen | 35-33 complex | unknown | • non-antigenic | [8] |
| Female-specific antigen | 28 | unknown | • antigenic | [8] |
| Actin fragments | 28-25 complex | Cytoskeleton, spines | • redox sensitive • polymeric  • antigenic | [8] [9] |
| Common antigens with schistosomula | 28-33 | Induce lethal anti-schla antibodies (in vitro) | • antigenic | [10] |
| Male-specific antigen | 20 | Unknown | • redox sensitive • antigenic | [8] |
| Female-specific antigen | 9 | Unknown | • redox sensitive • antigenic | [8] |
