# Supplementary material for: Immunoprotection of Mice against Schistosomiasis Mansoni Using Solubilized Membrane Antigens
Source: PLoS Negl Trop Dis. 2013 Jun 20;7(6):e2254. doi: 10.1371/journal.pntd.0002254 (PMC3688544; doi:10.1371/journal.pntd.0002254)
Supplement: References S1 — References to data and contents reported in Tables S1–3 are included in this section. (DOC) [file pntd.0002254.s004.doc]

**References S1**

[1] Sm97 (paramyosin): Pearce EJ, James SL, Hieny S, Lanar DE, Sher A (1988) Induction of protective immunity against *Schistosoma mansoni* by vaccination with schistosome paramyosin (Sm97), a nonsurface parasite antigen. Proc Natl Acad Sci U S A. 1988. 85: 5678-82. X; [2] Sm28 (Glutathione S-Transferase): [Balloul JM](http://www.ncbi.nlm.nih.gov/sites/entrez?Db=pubmed&Cmd=Search&Term="Balloul JM"%5BAuthor%5D&itool=EntrezSystem2.PEntrez.Pubmed.Pubmed_ResultsPanel.Pubmed_DiscoveryPanel.Pubmed_RVAbstractPlus), [Grzych JM](http://www.ncbi.nlm.nih.gov/sites/entrez?Db=pubmed&Cmd=Search&Term="Grzych JM"%5BAuthor%5D&itool=EntrezSystem2.PEntrez.Pubmed.Pubmed_ResultsPanel.Pubmed_DiscoveryPanel.Pubmed_RVAbstractPlus), [Pierce RJ](http://www.ncbi.nlm.nih.gov/sites/entrez?Db=pubmed&Cmd=Search&Term="Pierce RJ"%5BAuthor%5D&itool=EntrezSystem2.PEntrez.Pubmed.Pubmed_ResultsPanel.Pubmed_DiscoveryPanel.Pubmed_RVAbstractPlus), [Capron A](http://www.ncbi.nlm.nih.gov/sites/entrez?Db=pubmed&Cmd=Search&Term="Capron A"%5BAuthor%5D&itool=EntrezSystem2.PEntrez.Pubmed.Pubmed_ResultsPanel.Pubmed_DiscoveryPanel.Pubmed_RVAbstractPlus) (1987) A purified 28,000 dalton protein from *Schistosoma mansoni* adult worms protects rats and mice against experimental schistosomiasis. [J Immunol.](javascript:AL_get(this, 'jour', 'J Immunol.');) 15:3448-53. [3] Sm32 (Asparaginyl endopeptidase): Chacón N, Losada S, Bermúdez H, Cesari IM, Hoebeke J, Noya O (2003). [Immunogenicity of polymerizable synthetic peptides derived from a vaccine candidate against schistosomiasis: the asparaginyl endopeptidase (Sm32).](http://www.ncbi.nlm.nih.gov/pubmed/12941479?ordinalpos=1&itool=EntrezSystem2.PEntrez.Pubmed.Pubmed_ResultsPanel.Pubmed_DefaultReportPanel.Pubmed_RVDocSum) Immunol Lett. 8:199-210. [4] Sm31 (Cathepsin B): Klinkert MQ, Felleisen R, Link G, Ruppel A, Beck E (1989) Primary structures of Sm31/32 diagnostic proteins of *Schistosoma mansoni* and their identification as proteases. Mol Biochem Parasitol. 33:113-22. [5] Sm28-TPI (triosephosphate isomerase): Reynolds SR, Dahl CE, Harn DA (1994) T and B epitope determination and analysis of multiple antigenic peptides for the *Schistosoma mansoni* experimental vaccine triose-phosphate isomerase. J Immunol. 152:193-00.

**Reference S2**

[1] Oliveira G.C., Kemp W.M. (1995). Cloning of two actin genes from *Schistosoma mansoni*. Mol. Biochem. Parasitol. 75:119-122.

**References S3**

[1] Payares G, Smithers SR, Evans WH (1984) Purification and topographical location of tegumental alkaline phosphatase from adult *Schistosoma mansoni*. Mol Biochem Parasitol. 13:343-360. [2] Pujol FH, Cesari IM (1990) Antigenicity of adult *Schistosoma mansoni* alkaline phosphatase. Parasite Immunol 12:189-198. [3] Sauma SY, Strand M (1990). Mol Bioch Parasitol 38: 199-210. [4] Cesari IM, Bouty I, Alarcón de Noya B, Hoebeke J, Bout D (1992) Parasite enzymes as tool to investigate immune responses. In: Mem Inst Oswaldo Cruz 87 (Supl. IV): 55-65. [5] [Talla E](http://www.uniprot.org/uniprot/?query=author:"Talla+E."), [de Mendonca RL](http://www.uniprot.org/uniprot/?query=author:"de+Mendonca+R.L."), [Degand I](http://www.uniprot.org/uniprot/?query=author:"Degand+I."), [Goffeau A](http://www.uniprot.org/uniprot/?query=author:"Goffeau+A."), [Ghislain M](http://www.uniprot.org/uniprot/?query=author:"Ghislain+M.") (1998). *Schistosoma mansoni* Ca^2+^-ATPase SMA2 restores viability to yeast Ca^2+^-ATPase-deficient strains and functions in calcineurin-mediated Ca^2+^ tolerance. [J Biol Chem 273:27831-27840.](http://dx.doi.org/10.1074/jbc.273.43.27831) [6] Arnon R, Silman I, Tarrab-Hazdai R (1999) Acetylcholinesterase of *Schistosoma mansoni* - Functional correlates. [Protein Science](http://journals.cambridge.org/action/displayJournal?jid=PRS) 8(12): 2553-2561. [7] [Bhardwaj R](http://www.uniprot.org/uniprot/?query=author:"Bhardwaj+R."), [Krautz-Peterson G](http://www.uniprot.org/uniprot/?query=author:"Krautz-Peterson+G."), [Da'dara A](http://www.uniprot.org/uniprot/?query=author:"Da'dara+A."), [Tzipori S](http://www.uniprot.org/uniprot/?query=author:"Tzipori+S."), [Skelly PJ](http://www.uniprot.org/uniprot/?query=author:"Skelly+P.J.") (2011) Tegumental phosphosdiesterase SmNPP-5 is a virulence factor for schistosomes. [Infect Immun 79: 4276-4284](http://dx.doi.org/10.1128/IAI.05431-11). [8] unpublished. [9] this work. [10] Sher A, Kusel JR, Pérez H, Clegg JA (1974) Partial isolation of a membrane antigen which induces de formation of antibodies lethal to schistosomes cultured in vitro. Clin exp Immunol 18: 357-369.
